# Data driven polypharmacological drug design for lung cancer: analyses for targeting ALK, MET, and EGFR

**DOI:** 10.1186/s13321-017-0229-8

**Published:** 2017-07-04

**Authors:** Dilip Narayanan, Osman A. B. S. M. Gani, Franz X. E. Gruber, Richard A. Engh

**Affiliations:** The Norwegian Structural Biology Center, Department of Chemistry, Faculty of Science, UiT The Arctic University of Norway, Tromsø, Norway

**Keywords:** Lung cancer, Structure based drug design, ALK, MET, EGFR, Protein kinase inhibitor, Protein flexibility

## Abstract

Drug design of protein kinase inhibitors is now greatly enabled by thousands of publicly available X-ray structures, extensive ligand binding data, and optimized scaffolds coming off patent. The extensive data begin to enable design against a spectrum of targets (polypharmacology); however, the data also reveal heterogeneities of structure, subtleties of chemical interactions, and apparent inconsistencies between diverse data types. As a result, incorporation of all relevant data requires expert choices to combine computational and informatics methods, along with human insight. Here we consider polypharmacological targeting of protein kinases ALK, MET, and EGFR (and its drug resistant mutant T790M) in non small cell lung cancer as an example. Both EGFR and ALK represent sources of primary oncogenic lesions, while drug resistance arises from MET amplification and EGFR mutation. A drug which inhibits these targets will expand relevant patient populations and forestall drug resistance. Crizotinib co-targets ALK and MET. Analysis of the crystal structures reveals few shared interaction types, highlighting proton-arene and key CH–O hydrogen bonding interactions. These are not typically encoded into molecular mechanics force fields. Cheminformatics analyses of binding data show EGFR to be dissimilar to ALK and MET, but its structure shows how it may be co-targeted with the addition of a covalent trap. This suggests a strategy for the design of a focussed chemical library based on a pan-kinome scaffold. Tests of model compounds show these to be compatible with the goal of ALK, MET, and EGFR polypharmacology.

**Electronic supplementary material:**

The online version of this article (doi:10.1186/s13321-017-0229-8) contains supplementary material, which is available to authorized users.

## Background

The importance and proven druggability of protein kinases as targets in cancer [1, 2], inflammation [3], and other disease areas have transformed antikinase drug discovery into an information driven research area of unprecedented scale [4]. Public and proprietary databases contain binding data for hundreds of thousands of active compounds [5]. Crystal structures are publicly available for some 3000 protein kinase inhibitor complexes in the Protein Database (www.rcsb.org) [6]. This data begins to enable “polypharmacological” targeting of multiple kinases [7–9], which may more effectively modify network behavior [10], or forestall drug resistance [11, 12], or provide broader applicability against heterogeneous cancers or larger patient groups. Such approaches [13] may involve “retargeting” via modification of known compounds, or simply “repurposing” known compounds to new applications when target profiles are suitable. Practical approaches to polypharmacological design include both experimental and computational methods [14–18]. There is however no single strategic approach to modify such starting compounds to achieve the final selectivity profile; this depends on the availability, identification and understanding of the essential selectivity determinants of the relevant targets, as we examine with example of this paper.

**Fig. 1:**
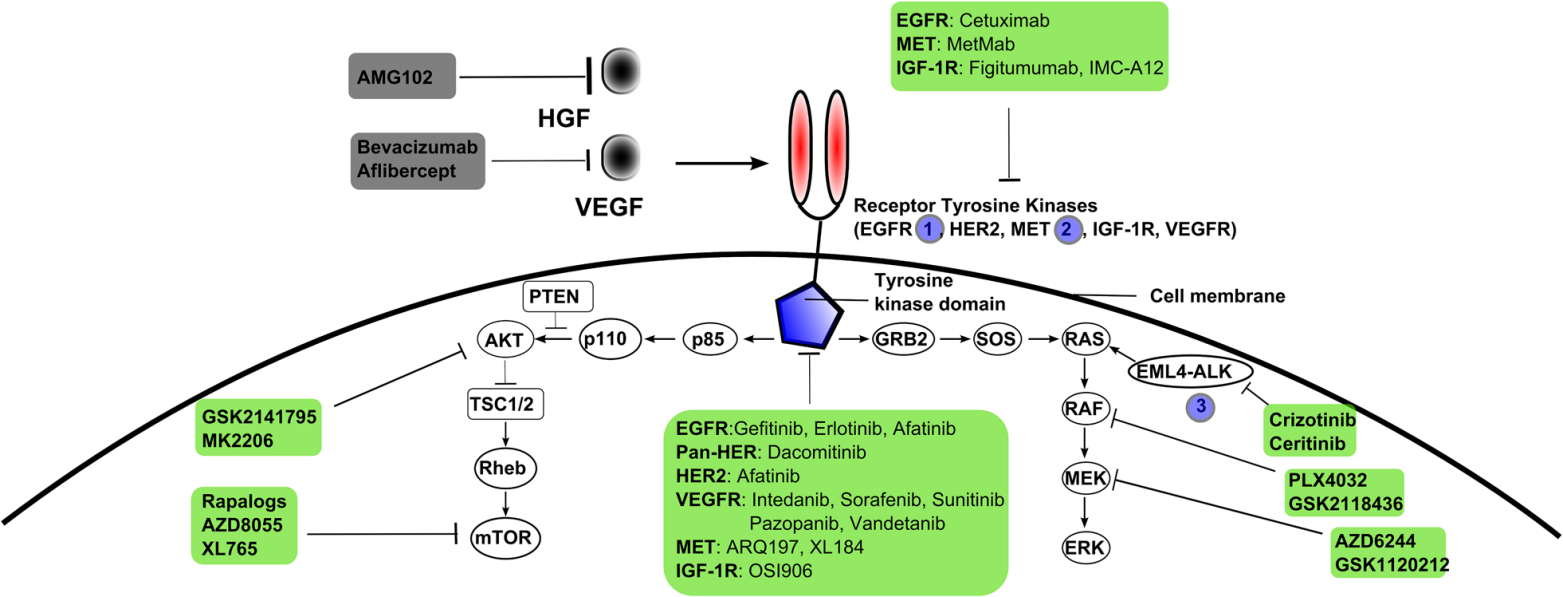
Overview of current molecular targets in advanced/metastatic non-small-cell lung cancer (NSCLC). Protein kinase inhibitors for NSCLC therapy, either approved or in advanced clinical trials, are shown in *green boxes*; antibodies against cytokine targets HGF asnd VEGF are *gray*. EGFR, MET and ALK are labeled with *blue circles*

Non-small-cell lung cancer (NSCLC) represents a collection of diverse molecular pathologies. Most types are relatively insensitive to chemotherapy, but the identification of genomic abnormalities in subpopulations of NSCLC patients [19–22] have led to the development of protein kinase inhibitors against EGFR [23, 24] (gefitinib, 2003; erlotinib, 2004; afatinib, 2013) and ALK (crizotinib, 2011; ceritinib, 2014; alectinib, 2015), see Fig. 1. These inhibitors specifically target either EGFR or ALK, but not both; cross-reactive inhibitors are under investigation however [25]. Analogous to the results of imatinib and ABL inhibition as therapy for CML, treatment with gefitinib and erlotinib is associated with acquired resistance [26]. Unlike ABL inhibition, resistance development to EGFR inhibitors seems universal. The most common resistance mechanism is a secondary mutation of the gatekeeper residue (for EGFR, predominantly T790M); afatinib appears less [27] but not completely [28] insensitive to this mutation. Additional mechanisms of acquired resistance include the amplification of MET [29], HGF [30], or HER2 [31]. The universal appearance of drug resistance via diverse mechanisms following EGFR inhibition therapy has generated widespread interest in polypharmacological or combinatorial treatment strategies [32–34]. In this paper we examine the potential of polypharmacological targeting EGFR, ALK, and MET.

As is typical for protein kinase inhibitors, compounds known to inhibit ALK, MET, and EGFR most potently bind at the ATP site, where they are anchored to the interdomain hinge via one or more hydrogen bonds [35]. Together, they represent a small subset of the known scaffolds for hinge/ATP site binders [36], which are already a restricted set [37] compared to proposed extents of possible scaffold diversity [38]. The EGFR inhibitor lapatinib was one of the first nearly monospecific kinase inhibitors [39]; others may have much broader selectivity profiles [40]. Knowledge of the determinants of selectivity for specific protein kinases or subfamilies [41, 42] assists target specific or polypharmacological drug design. These include the “gatekeeper” residue at the hinge, and infrequent occurrences of unique residues, such as glycine [43] or cysteine [44, 45]. The ongoing expansion of public [40, 46–49] and proprietary [50] target-ligand binding data begins to enable “machine learning” prediction of target inhibition profiles [51–56]. Such methods, similar to the more common structure based methods [57, 58], generally do not allow precise (e.g. binding constant error <10-fold) prediction of binding properties of individual compound-target interactions, but they do provide guidance to focus efforts on compound classes or libraries with the best chances for success [59–64].

In addition to the currently approved drugs and known inhibitors, many crystal structures are available to support drug design against NSCLC targets [6]. For EGFR, ALK, and MET, truncated kinase domain structures have been determined many times, uncomplexed, in complexes with ATP, ATP analogs, and inhibitors, including mutants and truncation variants. These structures show considerable diversity of active and inactive conformations [11, 13], and increasingly enable sophisticated drug design approaches. The target EGFR has become the primary example for targeting cysteine residues for irreversible inhibition [44, 45, 65] and, along with ABL, for the design of broadened selectivity profiles to overcome or forestall drug resistance [66]. A growing catalog of resistance mutants appearing in ALK [67–69] and MET also belong to the set of targets to be considered in general polypharmacological targeting strategies against NSCLC [70–78].

In this paper, we examine combinations of structure, binding, and target validation data to suggest a strategy for polypharmacological targeting of ALK, MET, and EGFR. We use cheminformatics methods to analyse the similarities of the targets. Inhibitor binding data shows a high degree of correlation between ALK and MET with respect to inhibition profiles, but also an essential dissimilarity with EGFR. The drug resistant mutant EGFR-T790M is intermediate between the two groups. We compare crystal structures of ALK and MET, considering especially the cross-inhibitory compound crizotinib, in an attempt to identify the structural origins of the similarities. Despite the dissimilarity of EGFR, the availability of a cysteine residue in the ATP pocket provides an orthogonal approach for polypharmacological optimization: the ALK-MET similarity may be exploited for optimization, while the addition of a covalent trap to inhibitors may add effective EGFR inhibition to the profile. Finally, we test compounds synthesized with these properties to verify the approach.

## Results and discussion

### Cheminformatics show similarities of ALK and MET, dissimilarity of EGFR, and intermediate similarity of EGFR mutant T790M

**Fig. 2:**
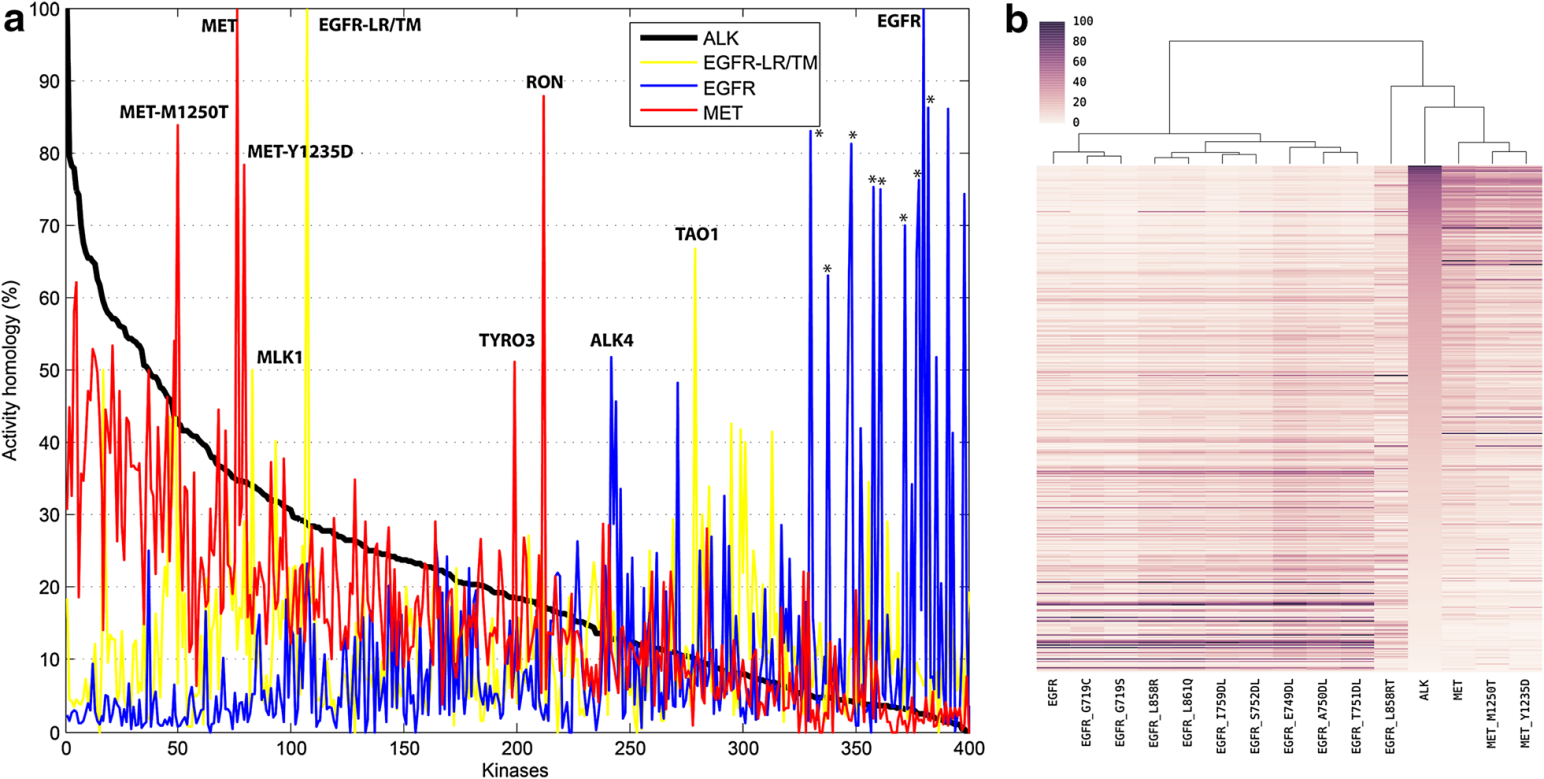
The “activity homology” (AH) similarity measure [80] as applied to ALK, MET, and EGFR. **a** Fractions of the sets of tight binding compounds of a reference PK target that also tighly bind to the tested PK are plotted for the ca 400 PKs of the test set. The curves are color coded according to the reference PK “A”: *black* for ALK, *red* for Met, *blue* for EGFR, and *yellow* for the drug resistant mutant EGFR-(L858R, T790M), which is abbreviated EGFR-LR/TM on the plot. The PKs of the test set are ordered according to the AH with ALK. Thus, Met-M1250T has the highest ALK AH (*black curve*) of the three Met variants (45%), EGFR is low (<5%), while EGFR-(L858R, T790M) is relatively high (30%). RON has the highest AH to MET, while TAO1 has the highest AH with EGFR-LR/TM. The peaks with high homology to EGFR marked with an *asterisk* are EGFR mutants other than EGFR-LR/TM, and have high AH similarity to EGFR (but not EGFR-LR/TM). **b** The same data, shown as a heirarchically clustered heat map. The mutant labelled L858RT represents the EGFR-(L858R, T790M) mutant, and is more similar to Alk and Met than to the other EGFR forms

Several measures are available to evaluate the similarities of kinase drug targets [15, 46, 79–82], including sequence, structure, and inhibitor properties. For drug discovery purposes, experimental binding data regarding cross-reactivity of inhibitors may be the most relevant, although this data may be generated in different ways, with diversity arising from choice of binding or activity assays, conditions, target protein form, etc. Significant discrepancies between assay formats are to be expected [80, 83, 84] which are more critically reflected in disparities between in vitro measurement conditions and their applicability in vivo. Based on single concentration measurement data from Ambit BioSciences, including estimated IC50 values for the binding of >20,000 compounds to 300–400 protein kinases, researchers at Bristol-Myers Squibb evaluated inhibitor selectivity profiles with an “activity homology” (AH) score [80] (see “Methods”). By this measure, ALK and MET are the most similar of the kinases considered in this manuscript, while EGFR is distinctly different (Fig. 2). Of the 400 protein kinases in the test set, some 80 kinases are more similar to ALK than MET, including the gatekeeper mutant M1250T of MET (approximately at position 50 of the 400 kinases). About 35% of the potent ALK inhibitors are shared with the MET inhibitor set (and about 43% with MET-M1250T). Less than 5% of the ALK inhibitors bind potently to EGFR and its mutants, with the notable exception of the double mutant EGFR-(L858R, T790M). This combination of the cancer primary mutation L858R and drug resistance gatekeeper mutation T790M is potently inhibited by 30% of the ALK inhibitors. (Figure 2 shows ALK4 as similar to EGFR, sensitive to over 50% of the EGFR inhibitors. This kinase “Activin-Like receptor Kinase” belongs to the Tyrosine Kinase Like (TKL) subfamily of the kinome, and is not related to ALK “Anaplastic Lymphoma Kinase” studied in this work).

**Fig. 3:**
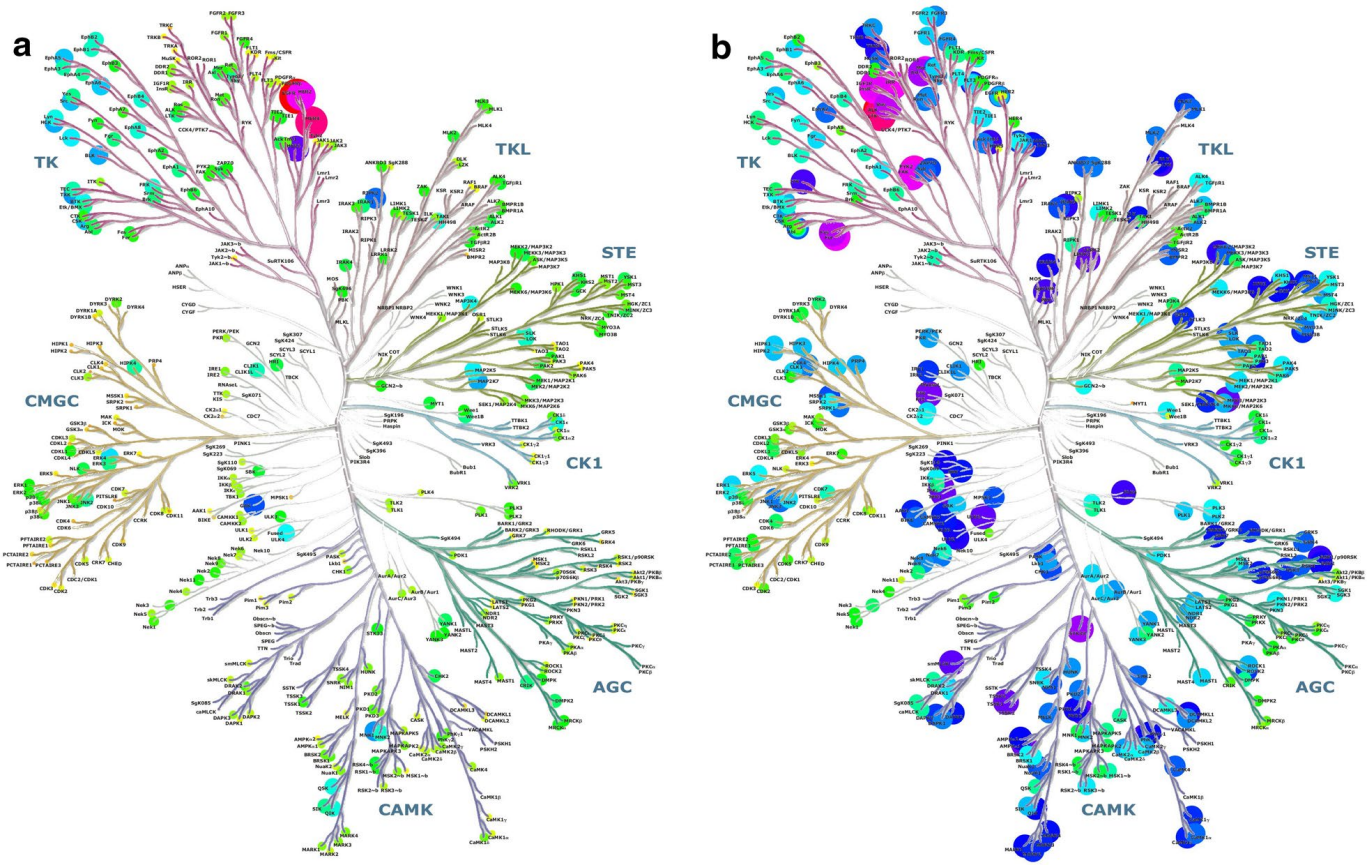
Correlations of inhibition profiles of the Ambit 2011 kinase profiling dataset [40]. Disk sizes and colors (*red* 100%, *magenta* 80%, *blue* 50%, *green* 20%) show the correlations of inhibition profiles of individual PKs with that of the PK of interest. **a** Correlations with ALK. **b** Correlations with EGFR

A related measure of similarity that also uses inhibitor binding data is the correlations of inhibitor binding profiles between pairs of kinases. Highly correlated targets share similar sensitivities to changes in the inhibitors. Unlike the “activity homology” described above, correlation compares the pattern of variation of inhibition strengths, and not the absolute values. Thus, two kinases may have highly correlated inhibition profiles even if the inhibition pattern is significantly weaker for one kinase. This may occur, for example, if the overall shapes of the inhibitor binding sites are similar, but one of the kinases may lack one important binding feature. For drug polypharmacology design purposes, it may be advantageous to enhance recognition of correlated sensitivities to ligand variation. Using the binding data of the Ambit study of 72 inhibitors and their interactions with 442 kinases [40], correlation analysis highlights the similarity of ALK and MET, and the dissimilarity of EGFR (Fig. 3). The inhibitor set of the study shows a large number of protein kinases, widely distributed across the kinome, with moderate similarity to ALK. The kinases with the most correlated inhibition profiles are, like ALK, tyrosine kinases, and include the closely related LTK, INSR, IRR and IGF1R kinases, but also FAK, PYK2, FER, FES, MER, and AXL. MET is only moderately correlated, and EGFR has low correlation. Indeed, very few kinases are correlated with EGFR; of the test panel, only HER4 and HER2 are strongly correlated, while HER3, a few TKs, and the less related RIPK2 and GAK kinases show moderate correlation similarity.

**Fig. 4:**
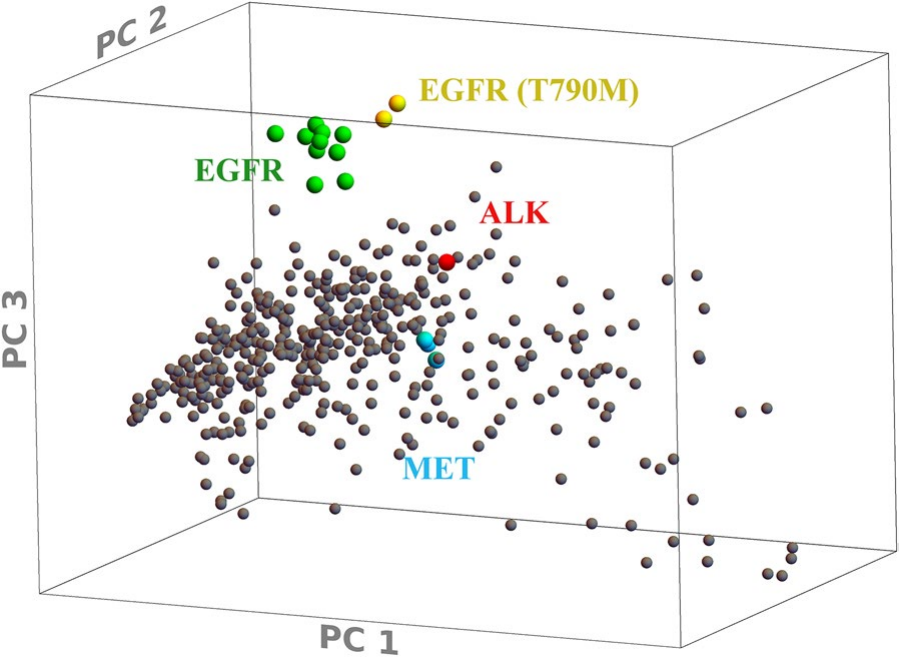
PCA transformation of the Ambit 2011 dataset, highlighting Alk, Met, and EGFR kinases. Of the first three dimensions of the principal component transformation of the dataset, prinicpal component #3 clearly distinguishes EGFR and variants from the other kinases, while ALK, MET, and EGFR all are similar with respect to PC #1. PC #2 distinguishes the T790M mutants from the other EGFR forms

A third measure of similarity is principle component analysis [85, 86] of multiple target-multiple inhibitor binding matrices (see “Methods”). Applied to the 2011 Ambit study [40], the protein kinase targets form a broad cluster, extended along the dimension of the first principal component (Fig. 4). Considering the PCA axes to represent “pseudo-inhibitors” as described above, there is a roughly Gaussian distribution for a majority of kinases around a value representing weak to moderate binding to “pseudo-inhibitor 1”, with some kinases in a skewed distribution toward tight binding. In order to maximize the variance in the first coordinate, the PCA method has constructed a “pseudo-inhibitor” that combines tight binding for the largest number of kinases possible (the skewed distribution). This favors the selection of targets which may be inhibited tightly by many inhibitors in the dataset, i.e. “generic” targets with high propensity for inhibition. ALK, MET, and EGFR are near the middle of the distribution in PC coordinate #1. The second PCA dimension similarly spreads the kinases into a moderately inhibited cluster, skewed toward a smaller set of kinases that are tightly inhibited; here,this includes EGFR and several of its variants, but not the two T790M mutants. The third PC dimension clearly separates EGFR and all of its mutants from the rest of the kinases, including ALK and MET. Thus, the activity homology (AH) data, the distributions of inhibitor correlation data on kinome plots, and PCA analysis of the inhibition data all show the statistical similarity of ALK and MET, the dissimilarity of EGFR, and an intermediate similarity for EGFR-T790M. The PCA analysis also identifies the inhibitors responsible for the clustering of EGFR and mutants away from the other kinases (see discussion below).

### Heterogeneity of crystal structures obscures polypharmacology prediction

Because the structures of the target proteins determine the binding strengths of the ligands, it is natural to expect crystal structures to reveal target similarities and to aid the formulation of polypharmacological targeting strategies. Accordingly, considering the previous section, the ALK and MET ATP binding sites would be expected to appear similar to each other than to EGFR, with the EGFR-T790M mutants somewhere in between. However, examination of the crystal structures available for these targets reveals more the difficulties of structure based prediction of comparative binding strengths than mechanisms for doing so. This is due in large part to the structural flexibility and plasticity of proteins, leading to signficant ligand induced structural changes, but also derives from structural distributions that are affected by the crystallization constructs, conditions and crystal packing arrangements. Considering all structures available for the targets illustrates this.

The ALK structures are most homogeneous set; they superimpose with an average Cα RMS of 0.11Å, have the same essential crystal packing arrangement and, with one exception (4FNY), share an active “DFG-in” conformation of the activation loop (with the DFG phenylalanine in its hydrophobic spine position [87]). The activation loop (A-loop) adopts a unique conformation, however: The A-loop phosphorylation site Tyr1278 is anchored below the “C-helix” on the side opposite to the ATP pocket, analogous to active TK structures first observed for insulin receptor kinase [88, 89], but in ALK with a unique alpha-helical secondary structure. The exceptional ALK DFG-out conformation structure shares the crystal packing arrangement of the other structures, but at a lower symmetry, with the asymmetric unit comprising what was a crystallographic symmetry related pair in the other structures.

The greatest number of structures is available for EGFR (more than 100 PDB entries when including disease-related and other mutations). The largest group of these structures share an active conformation, whereby pairs of monomers related by a crystallographic three-fold symmetry operation (space group I23) form an “asymmetric dimer” that represents a structural model of the active form [90]. Other EGFR structures show variations in C-helix positions (in = active, out = inactive); one set of structures (e.g. 2JIU) has an asymmetric unit consisting of a dimer with an apparently active geometry. There are no observations of a “DFG-out” geometry among the EGFR structures. However, there are two clusters of conformations: one with the usual active DFG-in conformation that places the DFG Phe into the hydrophobic spine, and another conformation with altered main chain angles and position of the activation loop.

The MET structures show the greatest conformational diversity. Of the three kinases studied here, MET has crystallized in the largest number of space groups, and the N- and C-lobe “open-closed” variations are largest. The DFG states observed include DFG-in, DFG-out, and intermediate states. The C-helix is seen in both “in” and “out” geometries. The activation loop conformations are highly varied, including many that could not be resolved in the crystal structures. Many of the variations also involve inhibitor interactions, and one common inhibitor binding surface is quite conserved as aromatic, but is seen formed variously by three different residues with four or more distinct geometries.

### Do crizotinib co-crystal structures explain cross-reactivity and reveal ALK and MET similarities?

**Fig. 5:**
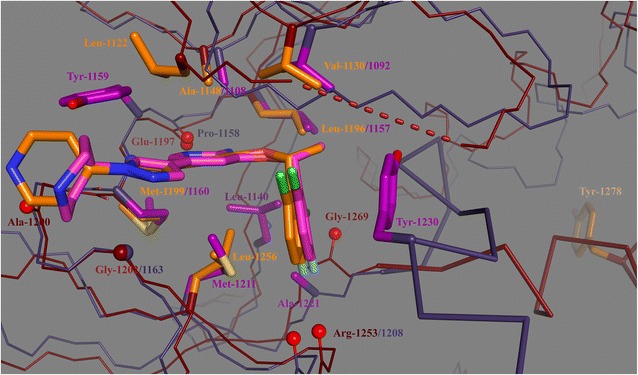
Superposition of structures of crizotinib in complexes with ALK (PDB: 2XP2; *orange*/*brick*) and MET (PDB: 2WGJ, *violet*/*indigo*). Side chains within a contact distance of 4 Å are shown as *sticks*, while main chain hydrogen bonding contact atoms are shown as *small spheres*. A *dashed line* indicates the approximate position of the disordered glycine-rich loop of Alk. The side chains of the activation loop phosphorylation sites are widely separated, with Tyr-1230 of MET in a pi–pi interaction with crizotinib, and Tyr-1278 of ALK anchored away from the ATP pocket by a helical conformation of the activation loop. The structure of crizotinib in complex with ALK mutant L1196M is similar to 2XP2, excepting the gatekeeper mutation

Even if flexibility prevents reliable prediction, it seems reasonable to expect that the structures of cross-reactive inhibitors in their different targets would identify the basis for the cross-reactivity. This would enable structure based design of e.g. an inhibitor library focussed on the likelihood of cross-reactivity. For ALK and MET cross reactivity, the low nanomolar inhibition of both ALK and MET by crizotinib is likely the clearest and best known measure of similarity between the two targets [91] Besides ALK (3 nM) and MET (2 nM), crizotinib also shows single digit nM binding (KD) to protein kinases ROS1 (4 nM), MER (4 nM), EPHB6 (6 nM), and AXL (8 nM) [40], depending of course on assay conditions. Crystal structures of crizotinib in protein kinases in the PDB comprise 2WGJ (c-MET KD), 2XP2 (ALK-KD), 2YFX (L1196M), 3ZBF (ROS1), 4ANQ (G1269A), 4ANS (L1196M, G1269A), and 4C9W (NUDT1). (The S-stereoisomer of crizotinib is co-crystallized with NUDT1 also). Superposition of co-crystal structures of crizotinib with ALK (2XP2 [92]) and MET (2WGJ [91]) reveals more how the binding energies that correspond to the highly selective and nanomolar ALK and MET co-inhibition depend on interactions that are not readily identified with standard structural biology or bioinformatic methods (Fig. 5). Interacting side chains differ at many key sites, including: the residues that sandwich the adenine binding site (MET vs. Leu from the C-lobe, and Leu vs. Ile from the glycine-rich loop), the gatekeeper+2 (the site two residues C-terminal to the gatekeeper residue) aromatic/hydrophobic side chain at the hinge (Tyr vs Leu), and a key pi–pi stacking interaction with the activation loop phosphorylation site tyrosine that is observed only in the MET structure. Amino acid type specific crizotinib interactions that are shared between ALK and MET comprise the gatekeeper (Leu), the C-terminal ATP site anchor of the glycine-rich loop (Val), an alanine residue two positions N-terminal to the active site lysine, and a pyrazole–proton interaction at a gatekeeper+6 glycine residue. The importance of the gatekeeper and pyrazole-glycine interactions are highlighted by the occurrence of resistance via mutation at these sites [93]. While the shared interactions are consistent with the observed co-inhibition, these residues are highly conserved in the kinome, so that these interactions are not predictive of the selectivity of the co-inhibition. Other shared but non-residue specific interactions include the anchoring hydrogen bonds at the hinge, and a key interaction with a main chain carbonyl group. The latter forms a CH–O hydrogen bond from an aromatic ring hydrogen that has been polarized by the fluorine substituent at the adjacent site on the ring [92]. The pi–pi stacking interaction with Tyr1230 appears important for MET inhibition, but no comparable interaction is seen in the ALK structure. This difference was proposed to account in part for the tighter binding of crizotinib to MET vs ALK, along with differences of the backbone peptide orientation at G1269 (ALK) and A1221 (MET) [92]. Crizotinib binding to the MET mutant Y1230C is weakened 15-fold in a cellular assay [94], supporting the view that the pi stacking interaction in important in vivo. Taken together, these details indicate that the crystal structures would not have allowed the prediction of high affinity cross-reactivity, but that prior knowledge of the cross-reactivity enables the study of the structures to identify the key candidate interactions responsible for it.

### Do the crystal structures in general reveal polypharmacological potential?

The importance of the CH–O, CH-arene, and arene–arene interactions for the total binding energetics (and, ultimately, therapeutic properties) of crizotinib is not entirely clear, but they highlight the scoring and searching dilemma that prevents in silico methods to predict ligand binding properties from target structures: The level of theory and concomitant CPU power required for computation of such binding features to provide effective scoring prevents their prediction a priori if binding geometries are unknown. (For example, the strength of the pyrazole–proton interaction described above depends on the electron-richness of the pyrazole and on energetic penalties of rotation away from the energy minimum, phenomena requiring quantum mechanics level calculations for evaluation [92]). Comparisons across all PDB structures provide some measure of the range of structural variability, but do not enable the calculation of binding energies, while inhibitor binding studies provide averaged binding energies averaged over structural distributions in the assay environmental conditions. In the PDB, there are currently 51 MET and 36 ALK structures; of these, only 3 MET structures are of the phosphorylated protein. For crizotinib, both MET and ALK were nonphosphorylated forms of truncated kinase domains, and the co-crystal structures showed inactive geometries. (MET has significant residual activity when unphosphorylated, and is activated 160-fold upon autophosphorylation [94]). For receptor TKs, phosphorylation often occurs autocatalytically in *trans* as a consquence of ligand binding to extracellular domains and oligomerization [95]. The mechanism of activation of tyrosine kinases by phosphorylation of the activation loop is usually considered to involve destabilization of possibly rigid inactive states of the kinase.

**Fig. 6:**
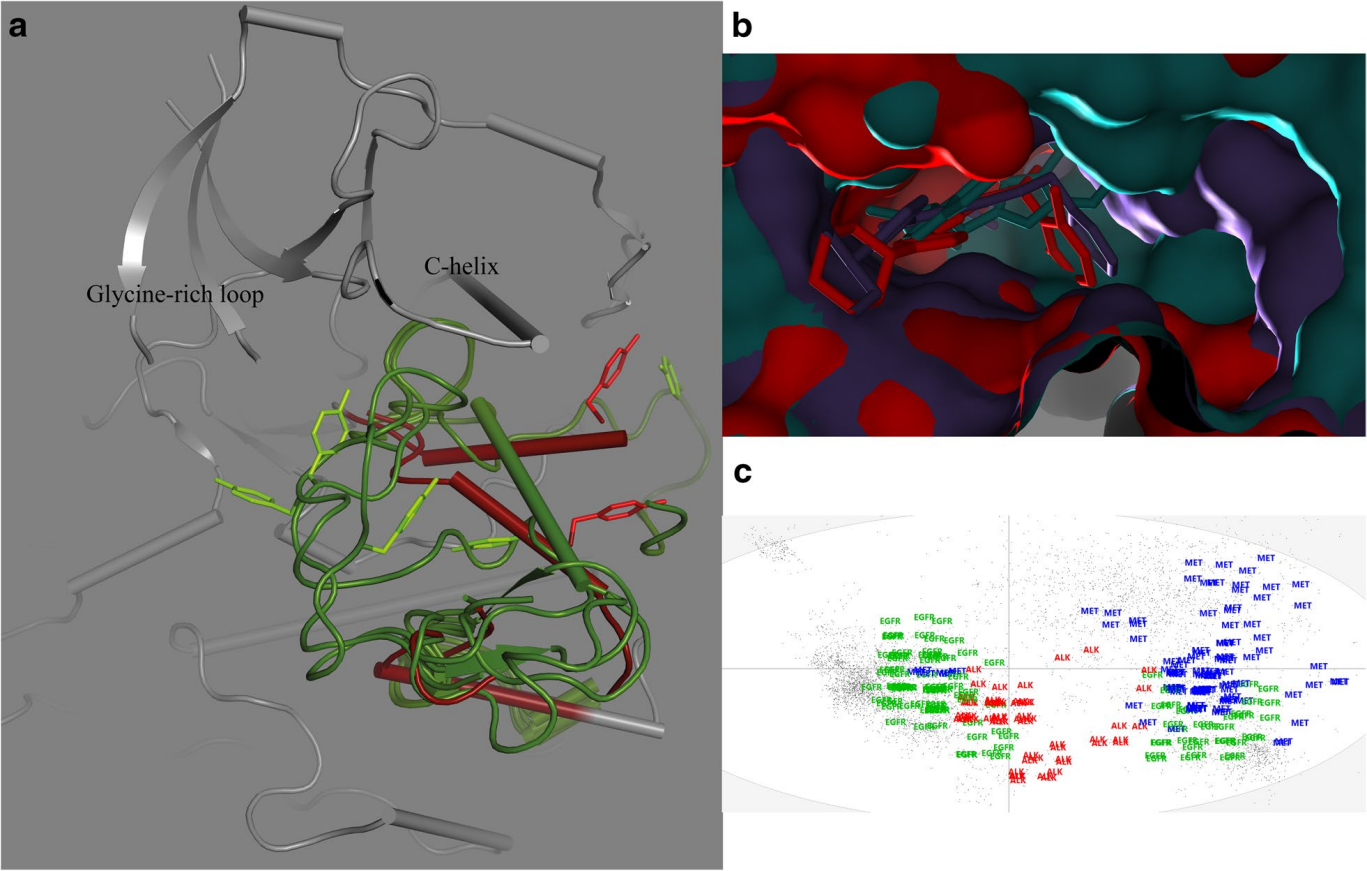
Structural variabilities and activation states of the targets. **a** A cartoon style plot showing the activation loops of representative crystal structures for MET (*green* PDBIDs 1R0P, 3DKC, 3F66, 3L8V, 3Q6W and 3RHK) and ALK (*red* both monomers from PDBID 4CNH) shows the diversity of positions for the tyrosine side chain suitable for crizotinib pi-stacking interactions (MET: Tyr1230, aligned position in ALK: Tyr1278). The ALK structures are two monomers from a single PDB structure that show different activation loop geometries. **b** Different shapes of the ATP binding sites arise from activation loop structural differences, among other differences. Here, structures for crizotinib-ALK (*red* PDBID: 2YFX), a pyrido-pyrimidine inhibitor and MET (*violet* PDBID: 4GG7), and ARQ197-MET (*turquoise* PDBID: 3RHK) are superimposed. **c** Distributions of activation relevant geometries for PDB structures of ALK, MET, and EGFR. The axes represent the first two dimensions of a PLS analysis to identify the geometric parameters most relevant to identify active and inactive states (see text)

Oncogenic activation via mutation or fusion often disrupts inactivating geometries. Partially as a result of this, and partially due to their inherent plasticity, protein kinase cancer drug target structures are highly flexible; prioritization for in silico drug discovery purposes may be difficult [96]. Superposition of ALK and MET structures (Fig. 6) from the PDB illustrate this. The MET structures show great diversity in conformations of the activation loop, and the tyrosine that is involved in pi–pi stacking interactions with crizotinib (Tyr1035) is found distributed across the entire space accessible to the activation loop (Fig. 6a). The structures for ALK are more homogeneous, and cluster into one major group and one minor group. Most ALK structures show a helical conformation for the activation loop following the DFG sequence, anchored to the C-helix by two arginine residues flanking the conserved glutamic acid of the C-helix, and by hydrophobic and aromatic interactions involving Tyr1278 (Fig. 6a). For the exceptional geometry, the activation loop retains a helical conformation and salt bridge interactions with helix C, but the Tyr1278 anchoring is lost, and the helix is rotated away from the C-helix. Prediction of ATP site binding will usually depend critically on the choice of the “correct” target structure (Fig. 6b), but if the “correct” structure is induced by inhibitor, it will obviously not be available for a priori searches. Statistical analysis of the protein kinases currently in the PDB provides an excellent illustration of the structural diversity currently observed to date [97]. PLS analysis [98] to identify the geometric measures that most strongly differentiate active and inactive geometries [97], and cluster crystal structures accordingly, shows how the structures for ALK, MET, and EGFR are distributed between apparent activity states. The MET structures are mostly inactive and broadly distributed, the ALK structures are mostly active (or close to it), and the EGFR structures are both active and inactive (Fig. 6c). For ALK, the position on the horizontal axis (with the coordinate definition dominated by DFG related geometries) does not clearly mark them as active. However, the vertical axis and its inclusion of helix C position parameters, coupled with “nearly active” DFG geometries, clusters ALK together with the active group.

**Fig. 7:**
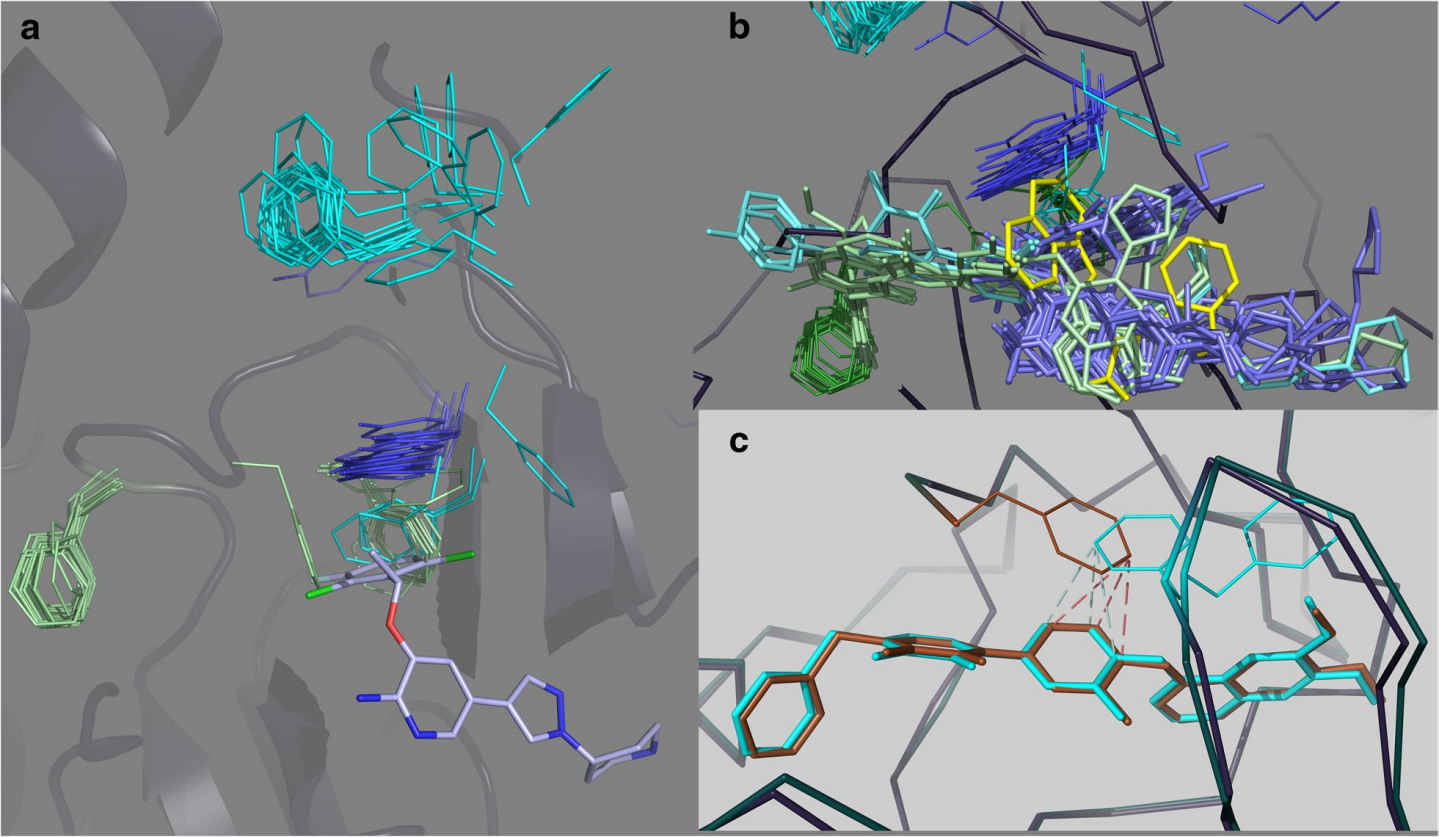
Variability of aromatic side chain positions. **a** A view (from the N-lobe side of the ATP pocket) showing clustering of aromatic amino acids. In MET structures, Phe1223 (*green*) of the “DFG” motif clusters into the DFG-in or DFG-out positions, with an intermediate position also represented. Phe1089 (*cyan*) from the glycine-rich loop of MET usually clusters near the tip of an extended glycine-rich loop, but is also seen in several structures to occupy nearly exactly the position adopted by the DFG Phe1223 in protein DFG-out configurations. Tyr1230, when interacting with inhibitors, forms a tight cluster at a single position, but is absent when Phe1089 or Phe1223 occupies an adjacent site. The PDBID codes for these structures are 2RFN, 2RFS, 2WD1, 2WGJ, 2WKM, 3A4P, 3C1X, 3CCN, 3CD8, 3CE3, 3CTH, 3CTJ, 3DKF, 3DKG, 3EFK, 3F66, 3F82, 3I5N, 3L8V, 3LQ8, 3Q6W, 3QTI, 3R7O, 3RHK, 3U6H, 3U6I, 3ZXZ, 3ZZE, 4AOI, 4AP7, 4DEG, 4DEH, 4DEI, 4EEV, 4GG5, 4GG7, and 2YFX. **b** Inhibitor types cluster according to the clustering of the target structures. Generally, type II inhibitors (*cyan*), with some diversity of chemical scaffolds, often bind in part via packing against Phe1223 in its typical DFG-out position, while type I inhibitors (*violet*) often bind via pi–pi stacking interactions against Tyr1230. The exceptional geometry of both protein and inhibitor for Arq197 (*yellow*) is apparent. The PDBID codes for these structures are: 2RFN, 2RFS, 2WD1, 2WGJ, 2WKM, 3A4P, 3C1X, 3CCN, 3CD8, 3CE3, 3CTH, 3CTJ, 3DKF, 3EFJ, 3EFK, 3F66, 3F82, 3I5N, 3L8V, 3LQ8, 3QTI, 3RHK, 3VW8, 3ZXZ, 3ZZE, 4AOI, 4AP7, 4DEG, 4DEH, 4DEI, 4EEV, 4GG5, and 4GG7. **c** The pyrimidone inhibitor of MET structure 3EFJ binds via pi–pi stacking interactions, but the dimer resolved in the* crystal* shows that the protein can provide the partner for this interactions with two different side chains, with identical inhibitor binding geometries

A closer look at the structural diversity reveals several interesting phenomena. One is the clustering of aromatic side chains at the ATP pocket (Fig. 7). Many of the MET structures show nearly identical positions for Tyr1230, similar to the geometry seen in the crizotinib complex. These are compatible with DFG-in geometries. Standard DFG-out geometries do not allow Tyr1230 to occupy that space, but replace it nearly exactly with Phe1223 of DFG, in place for inhibitor packing interactions. As a third alternative, this space may be occupied by the aromatic Phe1089 from the glycine-rich loop, represented by a small cluster of three structures in this superposition. There is also a unique positions for the DFG and glycine-rich loop positions. Inhibitor types are associated with the clusters of arenes at this site (Fig. 7b). Interactions with the DFG Phe in the DFG-out configurations are dominated by single aromatic rings from Type II inhibitors that occupy the deep pocket (vacated by the DFG-out Phe). Interactions with Tyr1230 of the activation loop involve a small variety of arenes: several halogenated or nitrated phenyl groups and a larger number of fused 5- and 6-membered heteroatomic aromatic ring systems, mostly in the same space. (One structure is displaced (3ZZE), but pi interactions may be maintained via resonance across nitrogen and amide bond linkages). One structure is unique: the complex with ARQ197 (PDBID: 3RHK [99]) shows a fused three-ring system sandwiched between Phe1089 and Phe1223 in unique positions. Finally, significant structural variation may be observed within a single crystal structure. The two monomers of the MET kinase domain in a crystal structure of a complex with a pyrimidone containing type II inhibitor (PDBID: 3EFJ [100]) shows that group to interact with the protein via pi–pi stacking interactions, whereby the interacting partner is the DFG Phe1223 for one monomer, but is the glycine-rich loop Phe1089 for the other (Fig. 7c).

Study of the variabilities shown by the crystal structures reveal physiologically relevant properties of the individual proteins and ligands studied, but their interpretation with respect to therapeutic properties requires much more experimental information and careful analyses of the differing environments *in crystallo* and in vivo. For protein kinases, crystal structures are commonly truncated single domain proteins with specific phosphorylation states, and the structures of flexible elements such as the activation loop may be determined by crystal packing interactions. In contrast, the disease targets are usually larger, multidomain proteins, often in larger assemblies, and with heterogeneities of chemical modifications and cellular environments. Highly potent inhibitors can compete with many of these effects, such that co-crystal structures generally reveal the key target-inhibitor interactions faithfully. But the crystal structures may also capture both the protein and ligand in states that are unimportant for therapeutic properties.

One uncertainty for protein kinase co-crystallography is the activation state of the enzyme. Crizotinib binding in MET described above involves pi–pi stacking with Tyr1230. However, the three activated MET structures in the PDB, phosphorylated on Tyr1235, show an activation loop structure with Tyr1230 far removed from the ATP binding pocket. The apparent structural homogeneity of ALK is also deceptive. The oncologically relevant forms of ALK are most commonly fusion proteins [76, 101] that remove membrane attachment and render ALK constitutively active. Dimer- or oligomerization is thereby essential for cell transformation, but the details of the structures and mechanism of activation are not known [76]. Mutations that confer resistance to crizotinib [102] include several that may destabilize the intramolecular A-loop helix packing that is apparently part of the inactivation mechanism for ALK [103]. The helix packs most prominently against the C-helix, with an Arg + Glu + Arg triplet, likely to stabilize a helical conformation, slotting into a space between two Glu residues extending from adjacent turns of the C-helix (of these, one is the usual partner to the active site lysine of active protein kinase structures. In addition, the phosphorylation site Tyr1278, which is adjacent to the anchoring Arg at the terminus of the activation loop helix, contributes to anchoring the helix via an edge-face pi–pi stacking interaction with Tyr1096.T his residue is found in a sequence N-terminal to the ALK kinase domain. The proximity of this anchor to the fusion position for the activation fusions with e.g. EML4- or NPM-suggest that the fusion may activate the protein by weakening the inactivating AL-helix interactions. For MET as a target in NSCLC, it is the wt protein which is of greatest relevance, although NSCLC related MET mutations have also been observed [104].

Summarizing the structures analysed here, we have seen most significantly: (1) that the diversity of activation forms of ALK, MET, and EGFR show how crystal structures cluster according to successful crystallization conditions, which is difficult to relate to the distribution of structures that are most relevant physiologically or biochemically (for in vitro binding measurements), (2) that knowledge of the cross-reactivity of crizotinib is a prerequisite for identifying key binding interactions, due to the divergence of sequences at the binding site, and (3) that these are most likely special interactions of arene groups and polarized bonds which would be overlooked by simplified molecular mechanics methods that are developed for rapid in silico approaches.

### How can cheminformatics inform crystallography?

It is clear that structures need to be interpreted with respect to binding data. Inhibition and/or binding data [47, 49] (including variation of ATP concentration and by single site mutation) are available from a variety of protein forms and assay formats [40, 82, 91, 92, 105–116]. Crizotinib binds to an inactive conformation of MET [94]. These data show insensitivity to phosphorylation in Abl, expected for type I inhibitors [117]). It is however affected by resistance mutations, whereby the T315I mutant of Abl is most tightly bound (ca 10 nM), and the gatekeeper+2 mutations F317I or F317L are most weakly inhibited (3000 and 600 nM, respectively). This sensitivity is interesting for considerations of ALK and MET binding, as described above. It should be readily appreciated that prediction of the ALK-MET cross-reactivity by automated bioinformatics or structural analysis methods seems highly unlikely. Whether more general machine-learning approaches could do so, presumably by identifying underlying and subtly interlinked selectivity determinants without recourse to model assumptions, remains to be seen. In any case, it will not be a competition between cheminformatics and crystallography, but will involve the integration of structural data into informatics techniques.

**Fig. 8:**
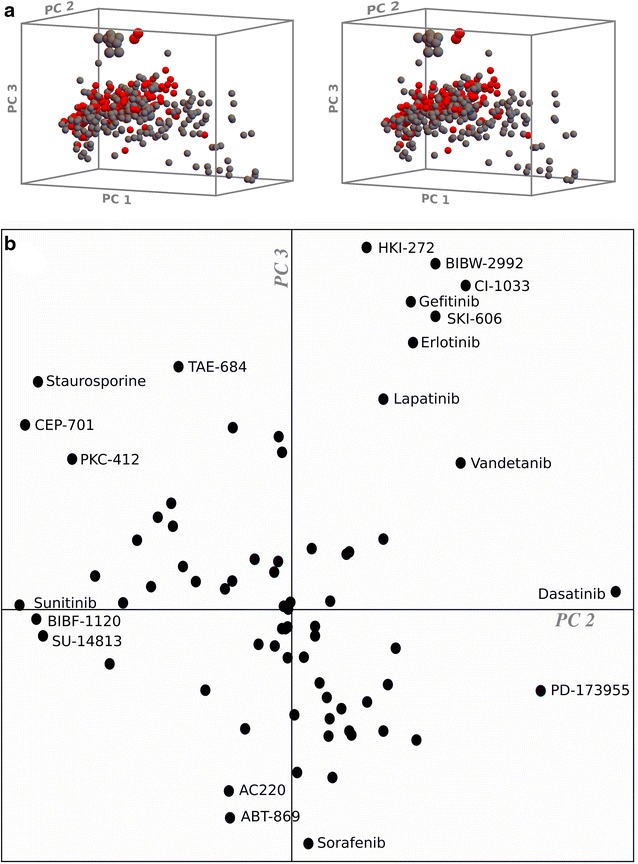
Does methionine as gatekeeper correlate generally with the selectivity properties of EGFR inhibitors? **a** Stereo plot of the first three PCA dimensions of the protein kinase inhibition data of the Ambit panel of 2011 (Fig. 4), with the protein kinases colored according to gatekeeper (*red* methionine, *gray* not methionine). EGFR and mutants are distinguished from the other kinases principally by PC axis 3, while the two gatekeeper mutant (T790M) forms of EGFR are distinguished from the other EGFR forms by PC axis 2. **b** The inhibitors contributing to the composition of PC axes 2 and 3 (loading plot) highlight the inhibitors that are potent for EGFR and most potent for the T790M mutations (*upper left*), those that are potent for EGFR but less potent for the T790M mutations (*upper right*), and inhibitors that are less potent or antiselective for EGFR (*lower two quadrants*)

Many statistical questions simply identify correlations, which may be recognized even with relatively sparse datasets. These may then generate hypotheses for more detailed study. The principal component analysis of the Ambit kinase and inhibitor panel of 2011 [40] generated transformed coordinates that indicated unique clustering for both EGFR and its drug resistant mutant T790M (Figs. 4, 8). The coordinate transformation highlights the inhibitors especially responsible for the unique position of EGFR. Early profiling data already indicated that EGFR could be targeted with unique selectivity [39]. The PCA transform of the 2011 data, and in particular the 2nd and 3rd dimensions, identifies inhibitors with particularly notable EGFR inhibition properties. PC dimension #2 (corresponding to a pseudo-inhibitor, see “Methods”), which separates EGFR T790M variants from the other EGFR mutants, also “bundles” selectivity determinants into the corresponding pseudo-inhibitor that have broad applicability to the rest of the kinome. One of these is most obviously the occurrence of methionine as gatekeeper, and the PCA plot shows the enrichment of protein kinase targets with this gatekeeper in the direction of the displacement of EGFR T790M variants relative to the remaining EGFR cluster. PC dimension #3, with less total variance than PC #2, has its clearest source of variance with the separation of all the EGFR targets. The “loading plot” for PC #2 and #3 (Fig. 8b) shows the inhibitors mostly responsible for the identification of these properties, and include highly selective EGFR inhibitors such as HKI-272, BIBW-2992, etc. (upper quadrants of Fig. 8b), and also antiselective inhibitors such as sorafenib (lower quadrants). Similarly, inhibitors that bind the native EGFR sequence preferentially (right-most quadrants, e.g. dasatinib) or the T790M mutant (left quadrants, e.g. staurosporine) are identified by PC dimension #2.

### Focussing libraries toward ALK + MET + EGFR polypharmacological inhibition

The analyses of this work reveal statistical target similarities between Alk and Met, along with a fundamental dissimilarity of EGFR, and an intermediate position for the drug resistant EGFR-T790M mutant. They also show however how distributions of structural variations, the importance of subtle chemical interactions, and mismatches between the systems used to generate experimental binding data prevent direct design of an inhibitor with the desired polypharmacological selectivity profile. As a consequence, the design goal is to create a library of test compounds with the greatest likelihood of having the target properties. For ALK, MET, and EGFR, the challenge is to achieve cross-reactivity despite the dissimilarity of EGFR. (The dissimilarity is shown statistically, as in Fig. 2, which also shows the existence of some overlap between potent inhibitor sets for ALK or MET and EGFR. One example inhibitor is brigatinib [116]). The solution is simple: use the known cross-reactivity of ALK and MET, which we may consider to be based on the “shape” of the respective ATP pockets, and add covalent trapping groups at sites with a good potential for reaction with the cysteine at the gatekeeper+7 site in EGFR.

**Fig. 9:**
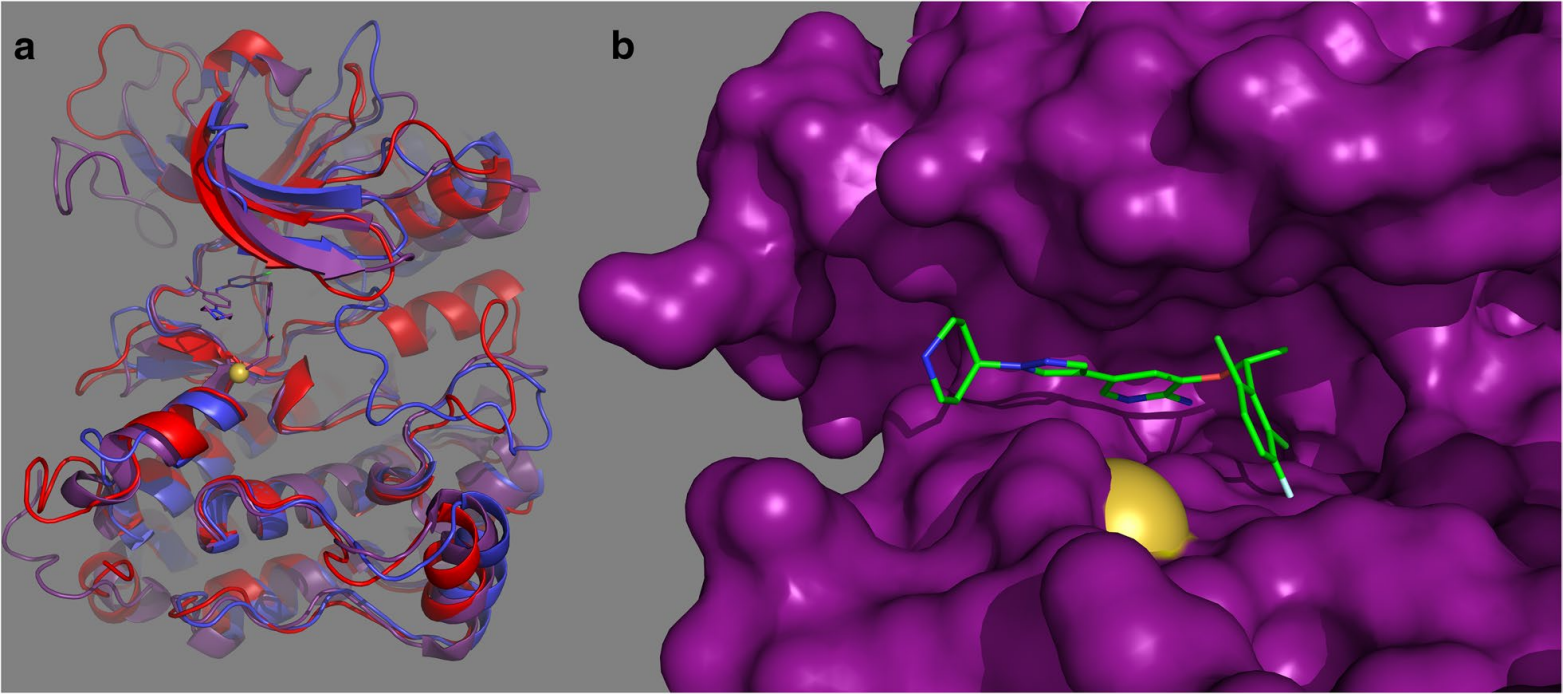
**a** Overlay of ALK (*red* PDBID: 3LCS), MET (*blue* PDBID: 2WKM), and EGFR (*magenta* PDBID: 3IKA) showing the relative positions of the tricycle inhibitor (here, from WZ4002 as stick model) and gatekeeper+7 targetable cysteine residue of EGFR. **b** Surface plot to show the relative positions of the gatekeeper+7 cysteine of EGFR (magenta surface) and crizotinib (modelled by superposition of the crizotinib-MET complex (PDBID: 2WGJ) with EGFR

Although the unusual nature of EGFR-inhibitor interactions revealed by the cheminformatics above does not depend on its cysteine at the gatekeeper+7 position, this cysteine does provide an ideal mechanism [45] for a high affinity interaction type that is essentially decoupled or “orthogonal” to shape-based similarity that characterizes ALK and MET. Thus, the detailed strategy to focus a library for ALK + MET + EGFR polypharmacological inhibition is first to identify compound classes that provide ALK + MET co-inhibition, and then to select scaffolds from these that in addition allow modification to target covalent inhibition of EGFR. The strength of the EGFR interaction need not be high, but would have to satisfy geometric and dynamic requirements for covalent binding. Many examples of suitable scaffolds have been published, and superpositions of the targets and relevant inhibitors (Fig. 9) show the viability of the approach.

**Fig. 10:**
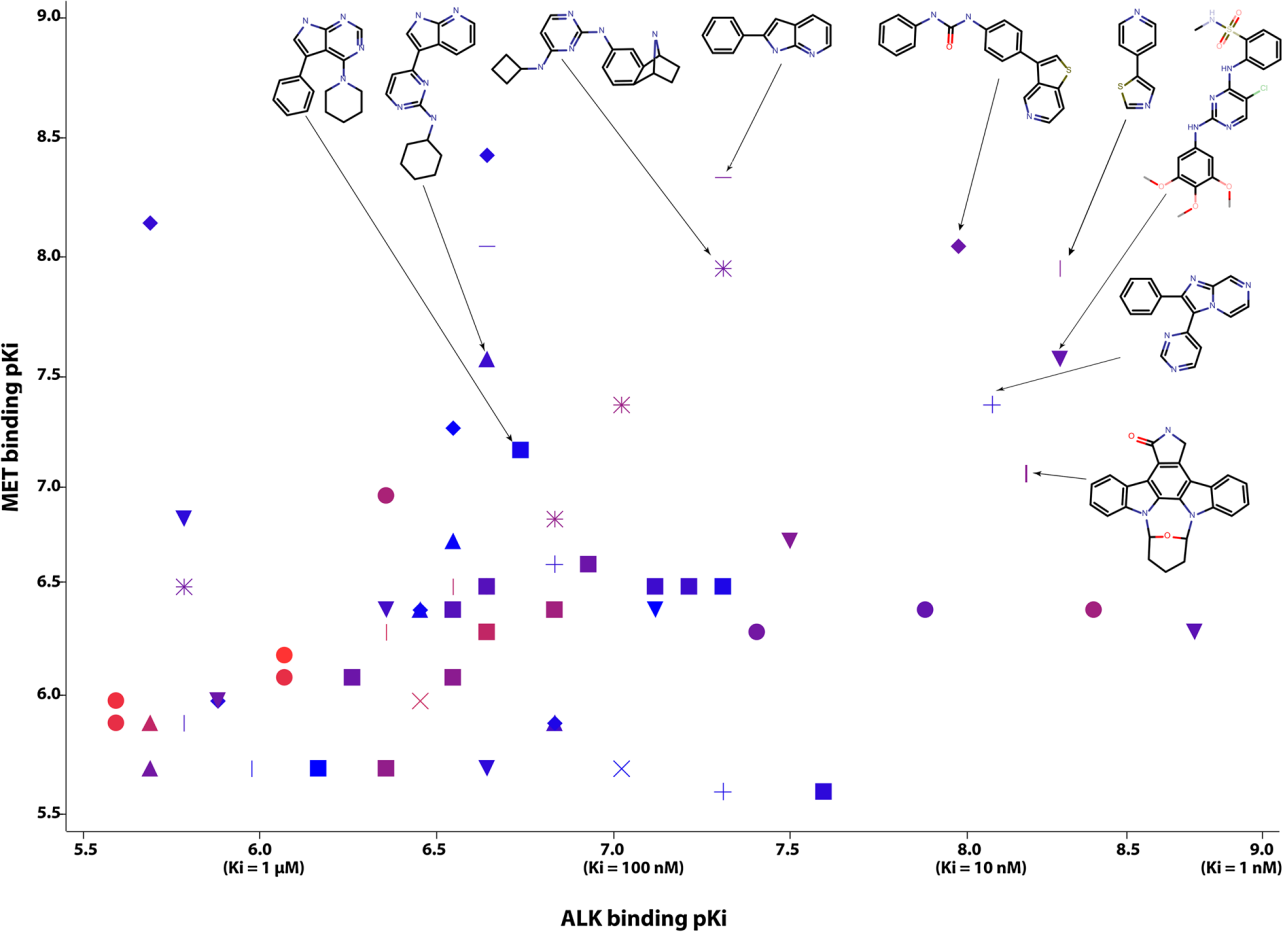
Candidate chemotypes for orthogonal EGFR covalent inhibition, prioritized based on the binding data of Abbott [46]. Values for individual inhibitors are plotted according to ALK and MET binding strengths, with chemotypes indicated by* symbol* (and defined for the tightest binders) and EGFR binding strengths indicated by color (*red* = 1 nM, *violet* = 10 nM, *blue* = 100 nM). The complete structure of the inhibitor for which the data point is plotted, is disclosed in the analysis [46], and the corresponding substituents are depicted for this chemotype in the figure at lower saturation

Crizotinib itself may be considered for this purpose, but it does not inhibit EGFR [40] (although is does bind the EGFR G719C mutant at micromolar levels). Other candidate scaffolds include for example (Fig. 10) staurosporine, bisindolylmaleimides, 4‐{2‐phenylimidazo [1,2‐a] pyrazin‐3‐yl} pyrimidine, and 3‐phenyl‐1‐(4‐{thieno[3,2‐c]pyridin‐3‐yl} phenyl) urea. Examination of the literature on candidate scaffolds and their suitability for ALK + MET + EGFR polypharmacology highlights especially the tricyclic scaffold found in the covalent EGFR inhibitor WZ4002 [118] (PDBID: 3IKA) and other derivatives known for covalent EGFR inhibition [119]. WZ4002 is known to bind both ALK [118] and MET [120], and the core dianilino-pyrimidine kinase binding scaffold also shows ALK and MET inhibition (Fig. 10) in other compounds [46]. This scaffold is well known in industry, including use as in connection with acrylamide groups for covalent binding, and a substructure search returns well over 104 compounds from patent literature. This need not hinder further research, however, because the earliest patents have expired or are due to expire soon (for example, methoxylated forms of (2,4) dianilino 5-chloropyrimidine were patented with a priority date of 1995 [121]).

**Fig. 11:**
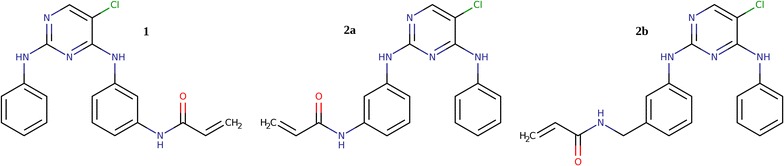
Tested compounds

 To test the suitability of dianilino-pyrimidine kinase binding scaffolds for ALK + MET + EGFR polypharmacology, we profiled three compounds representing the basic scaffold (including chlorine as the gatekeeper interacting atom: 5-chloro-N2,N4-diphenylpyrimidine-2,4-diamine), with additional single acrylamide functional groups as substituents on each of the candidate aromatic rings. Acrylamide substitution on the N4 phenyl group (at the *meta* position) places the covalent trap analogous to its position in WZ4002. Acrylamide substitution on the N2 phenyl moiety (also at the *meta* position) places the covalent trap at a position potentially suitable for a covalent trap to the gatekeeper+7 site in EGFR; varying the linker to the acrylamide function allows for uncertainties regarding optimal geometries and protein plasticity (Fig. 11; Additional file 1).

Tests of the compounds confirm suitability for ALK + MET + EGFR polypharmacology optimization (Table 1). For the N2 phenyl ring substituted compounds **2a** 1 and **2b,** 2 K_d_ values as measured by KdELECT assays (DiscoverX) show submicromolar binding for ALK, MET and both tested forms of EGFR. For compound **1,** 3 with the acrylamide function at the site corresponding to that of WZ4002, both ALK and MET binding were inhibited more weakly compared to EGFR binding. Retesting the compounds under scanKINETIC assay conditions for the two EGFR targets showed K_d_ values that were considerably tighter than in the KdELECT assays, approximately 2-fold for compound **2a**, and 4-7-fold for compounds **1** and **2b**. The sensitivity to the assay conditions seen for both forms of EGFR and uniformly tighter binding under the new assay conditions may indicate tighter binding for ALK and MET under these altered assay conditions as well.

**Table 1:** Alk, Met, and EGFR binding properties of the test tricyclic compounds

Compound	**1**	**2a**	**2b**
K_d_ study (nM)			
ALK	800	340	390
MET	>1000	250	330
EGFR^LR*^	420	710	780
EGFR^LR+TM§^	110	110	270
K_d_ values comparing 1 or 6 h incubation times (nM)^†^			
EGFR^LR^ (1 h)	57 (10%)	340 (14%)	170 (65%)
EGFR^LR+TM^ (1 h)	25 (13%)	56 (14%)	58 (170%)
EGFR^LR^ (6 h)	5.7	47	110
EGFR^LR+TM^ (6 h)	3.2	8	100
Apparent K_d_ values with 30× dilution after 1 h incubation (nM)^‡^			
EGFR^LR^ (30× dilution)	32 (56%)	340 (100%)	2900 (5.9%)
EGFR^LR+TM^ (30× diution)	12 (48%)	56 (100%)	4100 (1.4%)

^*^EGFR (L858R)

^§^EGFR (L858R, T790M)

^†^Percentages significantly lower than 100% indicate slow association

^‡^Percentages significantly higher than 3% indicate slow dissociation

The kinetics testing results (Table 1) support several further important conclusions. First, comparison of the the apparent binding constants comparing 1-h and 6-h incubation times show that compounds **1** and **2a** are relatively slow-binding, while binding of **2b** is complete within 1 h. Second, comparison of the apparent binding constants after 30-fold dilution following one-hour of incubation times showed slow dissociation behavior for compounds **1** and **2a**, and complete dissociation of **2b** within 5 h. The simultaneous appearance of slow association and slow dissociation complicates the interpretation of the results, but the percentage values shown in Table 1 show the apparent residual amounts of compounds relative to bound values at 1 h. These values indicate retention of roughly half of compound **1**, and nearly all of compound **2a**, after dilution. Covalent binding is the most obvious interpretation of these data. In contrast, compound **2b** follows fast association and dissociation kinetics, with no evidence of covalent binding. The similarity of compound **2a** to **2b** and **1** with respect to structure and binding strength, differing only in the linker to the acrylamide binding group, is further evidence that that it is the covalent binding property that determines the difference in binding kinetics, rather than e.g. a slow conformational change of the target enzymes.

The variations in properties of compounds **1, 2a** and **2b** indicate diversity of their potential application as basic scaffolds for generating libraries suitable for polypharmacological targeting of Alk, Met, and EGFR. Compound **1**, with analog WZ4002 known to bind covalently to EGFR, has an intrinsic selectivity for EGFR, but still may be suitable for Alk-Met-EGFR polypharmacology, with suitable decoration. Compounds **2a** and **2b**, on the other hand, show greater affinity for Alk and Met, and good affinities for both EGFR variants. Both **2a** and **2b** are thus good candidates for optimization. However, because **2a** and apparently not **2b** is able to bind covalently to EGFR (both forms), the **2a** scaffold seems most likely to provide good chances for polypharmacological optimization, as substituents may be chosen to optimize Alk and Met binding, using their intrinsically higher similarities, while maintaining EGFR binding via the covalent trap (providing high potency without the need for precise shape matching).

## Conclusions

The chain of reasoning of this work began with the identification of a set of relevant key targets for a disease, here non small cell lung cancer (NSCLC), and narrowing the selection based on the extent of available data on compounds and targets. Targets validated with approved inhibitors include EGFR, its drug resistant gatekeeper mutant of T190M, as well as ALK and MET. Mutations or fusions of both EGFR and ALK may be primary causes of cancer, whereas both the EGFR gatekeeper mutant T190M and MET may generate drug resistance. Analysis of the relevant inhibitor binding data identified similarities between ALK and MET, and highlighted the essential dissimilarity of EGFR, with an intermediate position for the T790M mutant. A result of this analysis is the choice of a strategy for polypharmacology to optimize ALK and MET binding via shape and surface complementarity, while maintaining covalent binding to EGFR. A suitable inhibitor scaffold was chosen, based solely on binding data. An accompanying analysis of the published crystal structures for the targets, aiming to guide optimization, highlighted mostly the intractability of mapping the inhibitor binding similarities to structural properites. This is due both to great structural variability of the targets and to the subtle nature of the essential interactions, which generally are not recognizable by molecular mechanics methods. However, the structures did highlight key details, such as the unusual binding interactions of crizotinib that are conserved between ALK and MET, despite differences in sequence, and also the binding mode of the dianilino-pyrimidine inhibitor scaffold. Thus, crystallography informs cheminformatics. This polypharmacology targeting example, while highlighting specific characteristics of ALK, MET, and EGFR, may be generalizable to other kinase target profiles in several ways. The flexibility and large numbes of kinases means that similar intractabilities of structural analysis will occur, but also that other combinations of diverse selectivity mechanisms may be found for appropriate targeting. Automation of such approaches seem highly unlikely until these mechanisms may be catalogued in some machine-analysable form. Other non-kinase polypharmacology approaches will likely be quite different, possibly involving target profiles more diverse, more rigid, or otherwise distinct from protein kinases.

## Methods

Activity homology data were taken from Posy et al. [80], with the data for Alk, Met, EGFR and EGFR-L858R, T790M plotted with Excel (Fig. 2a), and as a heat map (Fig. 2b) with tree clustering performed by the Clustermap function of the Seaborn python package (DOI: 10.5281/zenodo.45133). This measure is defined as the percentage of potent inhibitors (those with an estimated IC50 < 150 nM) of a reference kinase “A” that are also potent inhibitors of the comparison kinase “B”. As defined, this is “the prior probability that a compound will be active for kinase B given that it is active for kinase A” [80]. (Note that this defines a matrix which is not symmetric with respect to swapping the reference and compared kinases, because each will have a unique set of potent inhibitors.)

Kinome profiles of target similarity (Fig. 3) were evaluated using the data of Davis et al. [40], with the nM inhibition values x_i_ converted into logarithms to be proportional to binding energies for inhibition values tighter than the upper measurement limit of 10 micromolar, and arbitrarily set uniformly to 5 (corresponding to 100 micromolar) for values above the upper measurement limit. These values were used to calculate target similarity as Pearson’s correlation coefficient for the pairs of vectors of binding energy equivalents and plotted as disks at the kinase positions of the kinome plot of Manning et al. [122]. Hues and radii reflect the correlation values.

Principal components (Figs. 4, 8) were determined using Karhunen–Loeve Decomposition as implemented in Mathematica (version 10.3) with the binding energy equivalent values. This method of data analysis can be applied to an *t* · *n* matrix **T**_ij_ that represents how the *t* targets are inhibited by *n* inhibitors (the targets {i} are thus *t* positions in an *n*-dimensional space of inhibitors {j}). The analysis determines the orthonormal linear transformation of the coordinate system that diagonalizes the *t* · *t* covariance or correlation matrices of the targets with respect to their inhibition profiles. This transformation into a new *n* dimensional space of virtual or pseudo-inhibitors {k} is done (usually with the {T_i,1_, T_i,2_,…} vectors normalized to unit variance and zero average) such that the variance is maximal for k = 1 (PCA dimension 1), second highest for k = 2 (PCA dimension 2), and so on. This reduces the redundancy of similarities between inhibitors, and clusters the targets in spaces whose dimensionality may be reduced according to the degree of variance desired “to explain the data”. As the PCA transformation maximizes the spread in inhibition values by a “pseudo-inhibitor” created by the recombination of inhibitors of the dataset, inhibitors that are selective for subgroups of targets are most strongly represented in the relevant transformed coordinates, with the (mutually orthogonal) coordinates ranked (PC #1, PC #2, etc.) according to the total variance in that coordinate. The “loading plots” show the coefficients of the individual inhibitors in the “pseudo-inhibitor” principal components created by the transformation.

Superpositions of the ALK, MET, and EGFR protein kinase structures from the Protein Data Bank (PDB) (Additional file 2) were performed using PYMOL (version 1.7) and scripts that extracted protein kinase monomers from the entries. The scripts aligned the CA atoms from the gatekeeper+3 residue as hinge anchor position (1196–1199—ALK, 1158–1161—MET, 766–769 or 790–793—EGFR), along with aF helix atoms as the core of the C-lobe (1308–1324—ALK, 1262–1278—MET, 869-885 or 893–909—EGFR) residues.

SIMCA (v.13, Umetrics AB, Umeå, Sweden) was used to build a PLS regression plot [123] (Fib 6c) by taking 233 parameters (distances, angles, dihedrals etc.) from the annotated dataset of Möbitz [97] as independent variables and the designated active/inactive state of the kinase domains as the dependent variable. Thus, the PLS method transforms the dimensions of the individual structural parameters [97] into orthogonal dimensions that maximize covariance of the transformed dimensions with the activity state of the kinase. In the SIMCA implementation, a 7-fold cross validation technique prevents overfitting in the estimate of the number of significant components. The variables were scaled by unit variance. The PLS model built from this dataset has 4-components with both R2 (goodness of fit, maximum 1) and Q2 (predictive ability, maximum 1) values more than 0.9, where 1st and 2nd components account for about 80 and 6% of the variations respectively.

 The three tricyclic compounds (**1**, **2a** and **2b**) were purchased from the company ARTTSynthesis (www.arttsynthesis.com). Tests for binding properties were performed by the company DiscoverX (www.discoverx.com), using a concentration of 1 µM against ALK, MET and EGFR variants L858R and L858R_T790M) using KdELECT and scanKINETIC from DiscoverX [124, 125].

## Additional file

**Additional file 1: Compound_SMILES_strings.rtf.** SMILES strings for compounds tested in this manuscript (Figure 11).

**Additional file 2: PDBIDs-Used-in_analysis.** Summary of PDB codes used in the figures of this manuscript.

## Abbreviations

PK: protein kinases; this work cites ABL, ALK, AXL, EGFR, EPHB2, FAK, FER, FES, GAK, HER2, IGF1R, INSR, IRR, LTK, MER, MET, PYK2, ROS1, and RIPK2
EML4: echinoderm microtubule-associated protein-like4
HGF: hepatocyte growth factor
NPM: nucleophosmin
NUDT1: hydix hydrolase 1
TK: tyrosine kinase (protein kinase group)
TKL: tyrosine kinase like (protein kinase group)
AH: activity homology
ATP: adenosine triphosphate
CML: chronic myelogenous leukemia
DFG: Asp-Phe-Gly initiating sequence of the activation loop
NSCLC: nonsmall cell lung carcinoma
PC: principal component
PCA: principal component analysis
AL or A-loop: activation loop
PLS: projection on to latent spaces, or equivalently partial least squares
PDBID: Protein Data Bank ID code

## Footnotes

1 N‐(3‐{[5‐chloro‐4‐(phenylamino)pyrimidin‐2‐yl]amino}phenyl)prop‐2‐enamide.

2 N‐[(3‐{[5‐chloro‐4‐(phenylamino)pyrimidin‐2‐yl]amino}phenyl)methyl]prop‐2‐enamide.

3 N‐(3‐{[5‐chloro‐2‐(phenylamino)pyrimidin‐4‐yl]amino}phenyl)prop‐2‐enamide.
