# Supplementary material for: Data driven polypharmacological drug design for lung cancer: analyses for targeting ALK, MET, and EGFR
Source: J Cheminform. 2017 Jul 4;9:43. doi: 10.1186/s13321-017-0229-8 (PMC5496928; doi:10.1186/s13321-017-0229-8)
Supplement: Supplementary file 1 — Additional file 1: Compound_SMILES_strings.rtf. SMILES strings for compounds tested in this manuscript (Figure 11). [file 13321_2017_229_MOESM1_ESM.rtf]

cpd_1 = 'C=CC(=O)Nc3cccc(Nc2nc(Nc1ccccc1)ncc2Cl)c3'
cpd_2a ='C=CC(=O)Nc3cccc(Nc2ncc(Cl)c(Nc1ccccc1)n2)c3'
cpd_2b = 'C=CC(=C)NCc3cccc(Nc2ncc(Cl)c(Nc1ccccc1)n2)c3'
