# Supplementary material for: Data driven polypharmacological drug design for lung cancer: analyses for targeting ALK, MET, and EGFR
Source: J Cheminform. 2017 Jul 4;9:43. doi: 10.1186/s13321-017-0229-8 (PMC5496928; doi:10.1186/s13321-017-0229-8)
Supplement: Supplementary file 2 — Additional file 2: PDBIDs-Used-in_analysis. Summary of PDB codes used in the figures of this manuscript. [file 13321_2017_229_MOESM2_ESM.rtf]

PDB codes for structures used to prepare the figures

Figure 5: 2WGJ, 2YFX, 2XP2
Figure 6a: 1R0P, 3DKC, 3F66, 3L8V, 3Q6W and 3RHK and 4CNH
Figure 6b: 2YFX, 4GG7, 3RHK
Figure 7a: 2RFN,  2RFS,  2WD1,  2WGJ,  2WKM,  3A4P,  3C1X,  3CCN,  3CD8,  3CE3,  3CTH,  3CTJ,  3DKF,  3DKG,  3EFK,  3F66,  3F82,  3I5N,  3L8V,  3LQ8,  3Q6W,  3QTI,  3R7O,  3RHK,  3U6H,  3U6I,  3ZXZ,  3ZZE,  4AOI,  4AP7,  4DEG,  4DEH,  4DEI,  4EEV,  4GG5,  4GG7, 2YFX
Figure 7b: 2RFN, 2RFS, 2WD1, 2WGJ, 2WKM, 3A4P, 3C1X, 3CCN, 3CD8, 3CE3, 3CTH, 3CTJ, 3DKF, 3EFJ, 3EFK, 3F66, 3F82, 3I5N, 3L8V, 3LQ8, 3QTI, 3RHK, 3VW8, 3ZXZ, 3ZZE, 4AOI, 4AP7, 4DEG, 4DEH, 4DEI, 4EEV, 4GG5, 4GG7
Figure 7c: 3EFJ
Figure 9a: 3LCS, 2WKM, 3IKA
Figure 9b: 3IKA, 2WGJ
